# A Case of Granulomatosis with Polyangiitis (GPA) Where a Multicystic Nasal Septal Abscess Aided in the Diagnosis

**DOI:** 10.1155/2022/7415498

**Published:** 2022-10-12

**Authors:** Mayuko Sasawaki, Kazuhiro Omura, Teru Ebihara, Nobuyoshi Otori

**Affiliations:** ^1^Department of Otorhinolaryngology, The Jikei University School of Medicine, 3-25-8 Nishi-shinbashi, Minato-ku, Tokyo 105-8461, Japan; ^2^Department of Otorhinolaryngology, Dokkyo Medical University, Saitama Medical Center, 2-1-50, Minami-Koshigaya, Koshigaya, Saitama 343-8555, Japan

## Abstract

A 69-year-old male patient presented to the hospital with a chief complaint of nasal obstruction. Physical examination revealed swelling of the anterior nasal septum and nasal dorsum and tender indurated oedema of the dorsum of both hands. Blood tests showed an elevated inflammatory response, and contrast-enhanced computed tomography (CT) showed a polycystic abscess in the nasal septum. Emergency surgery and histopathology were performed on the day of the initial visit for incisional drainage. Intraoperative findings showed white necrosis between the nasal septal cartilage and nasal septal mucosa, as well as white necrosis and pus accumulation in the periosteum and soft tissue of the piriform aperture and the nasal bone. The patient underwent endoscopic dissection and drained as much as possible, and the abscess and surrounding normal nasal septal mucosa were sampled for diagnostic purposes. The patient was diagnosed with vasculitis based on the clinical findings, pathological examination results, and blood test results. After the diagnosis was confirmed, steroid and cyclophosphamide pulse administration was initiated, and the swelling of the anterior nasal septum and nasal dorsum and the bilateral dorsal indentation oedema improved markedly. The patient is now doing well and will continue to be carefully monitored in the outpatient clinic.

## 1. Introduction

Granulomatosis with polyangiitis (GPA), formerly known as Wegener's granulomatosis, is a systemic refractory vasculitis that presents with necrotising granulomatous inflammation in the upper respiratory tract and lungs, necrotising glomerulonephritis in the kidney, and necrotising vasculitis of medium and small arteries throughout the body. GPA is a systemic refractory vasculitis. Although the prognosis of this disease is poor, early diagnosis and therapeutic intervention have led to the remission of the disease in an increasing number of cases. Physical examination is important in the diagnosis of granulomatosis polyangiitis. Upper respiratory tract symptoms are often reported as saddle nose and perforation of the nasal septum, and only one case of the nasal septal abscess has been reported as an upper respiratory tract symptom.

Herein, we describe a case of GPA with the nasal septal abscess that resulted in a visit to our department. Since upper respiratory tract symptoms account for 70%–100% of all cases of GPA, a patient will likely visit an otorhinolaryngologist for the first time. The nasal cavity and paranasal sinuses are the most common sites involved in the head and neck region (85%–100%), but ear symptoms may develop initially in approximately 35% of cases, as discussed by Greco [1].

Otolaryngologists need to be aware of GPA, which often develops due to upper respiratory tract symptoms. However, the actual diagnosis is not easy because lesions confined to the upper respiratory tract alone often do not present with symptoms and findings typical of GPA.

There have been many reports of nasal involvement due to GPA, such as thickening of the mucosa of the nasal septum [2], perforation of the nasal septum [3], saddle nose due to nasal bone destruction [4], and chronic dacryocystitis and nasolacrimal duct stenosis [5]. However, there have been few reports of nasal septal abscesses, such as in our case. Moreover, there are few reports of nasal septal abscesses, and only two cases, including an autopsy, have been reported.

## 2. Case Report/Case Presentation

A 69-year-old male patient presented to the outpatient clinic of the Department of Otorhinolaryngology with a chief complaint of nasal obstruction persisting for 4 months. He had a history of diabetes mellitus and no history of rheumatoid arthritis.

**Figure 1:**
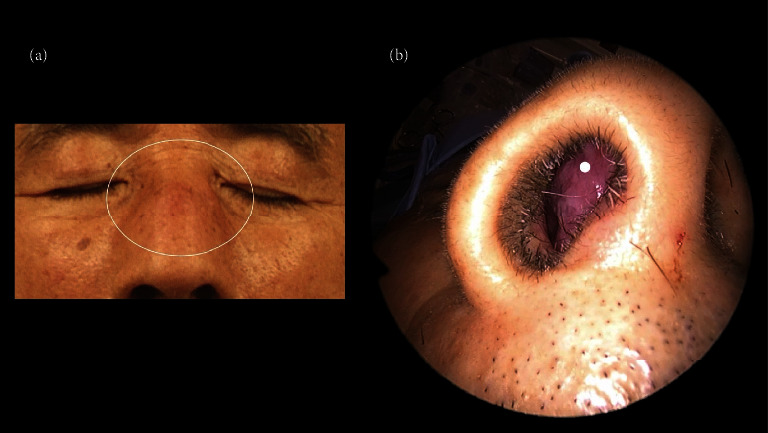
(a). Physical findings of the face showing a swelling of the dorsum of the nose (white circle). (b). Physical findings of the face showing a swelling of the anterior nasal septum of the nose (white dot).

Physical examination revealed swelling of the anterior nasal septum and dorsum of the nose (Figures 1(a)-1(b)).

**Figure 2:**
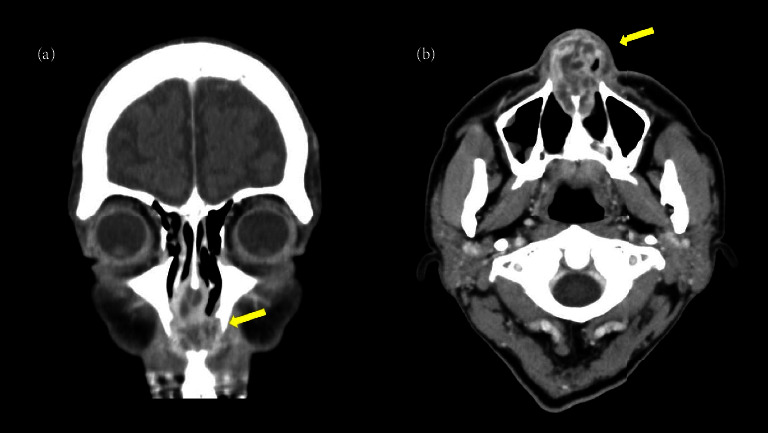
(a). Coronal contrast-enhanced CT of the paranasal sinuses showing multifocal abscess in the nasal septum (yellow arrow). (b). Axial contrast-enhanced CT of the paranasal sinuses showing multifocal abscess in the nasal septum (yellow arrow).

Blood tests showed that the white blood cell count was elevated to 15.8 × 10^3^, and C-reactive protein had increased to 11.76. Meanwhile, the creatinine level was in the normal range, and there were no proteinuria, haematuria, or rheumatological problems. Chest computed tomography (CT) also showed no abnormality. Contrast-enhanced CT showed multiple abscesses in the nasal septum (Figures 2(a)-2(b)).

Drainage surgery was performed in parallel with histopathological examination. Intraoperative findings showed white necrosis between the nasal septum cartilage and mucosa, as well as white necrosis and pus accumulation in the periosteum in the pear-shaped opening to the nasal bone. The mucosa of the nasal septum was incised and drained, and a biopsy of the mucosa in and surrounding the abscess was performed endoscopically (Video 1).

Bacterial abscess formation was suspected; therefore, antimicrobial agent therapy was initiated immediately after the surgery, albeit with no improvement. Subsequently, blood tests revealed a prolonged inflammatory response and nasal septal abscess culture tests were negative during hospitalisation. Further blood tests showed PR3-ANCA positivity. In addition, epithelial cell granuloma and vasculitis were discovered in the specimens collected during emergency surgery. From this information, we reached a consensus regarding our diagnosis. After confirming the prognosis, we immediately started the patient on steroids at 40 mg per day due to an early systemic form of granuloma, resulting in an improvement in the swelling of the anterior nasal septum and dorsum of the nose and the indented oedema of the dorsum of both hands, and the fever receded.

Consequently, intravenous cyclophosphamide 500 mg per 2 weeks was also added as remission induction therapy.

**Figure 3:**
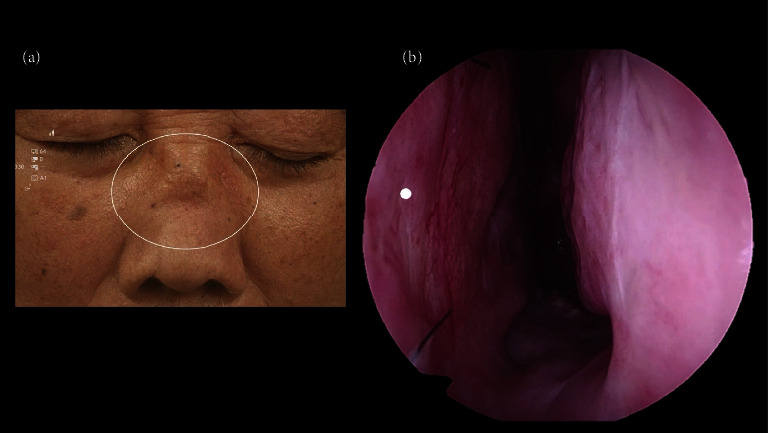
(a). Physical findings of the face showing a saddle nose (white circle), a typical physical finding of granulomatosis with GPA. (b). The 0° endoscopic findings of left nasal cavity. There is no nasal perforation after the surgery of biopsies from nasal septum (white dot).

In addition, blood tests revealed an improvement in the inflammatory response. One month after the surgery, the swelling of the external nose subsided, followed by a saddle nose, a typical physical finding of granulomatosis with GPA (Figures 3(a)-3(b)). The patient remains in good condition and is being carefully monitored in the outpatient clinic.

## 3. Discussion/Conclusions

The probability of obtaining characteristic histological findings on biopsy for GPA is only 40%–60% [2, 6]. Therefore, the following technique for biopsy tissue collection is required. Areas of strong ulceration or necrosis must be avoided, and a large sample of an adjacent undisturbed area must be taken (specifically, more than 5 mm in diameter) [7]. Samples from multiple sites must be taken and the procedure must be repeated if there is no specific finding [6].

When GPA is considered or suspected, an active biopsy must be performed [6, 8, 9].

In our case, a nasal septal abscess was the main symptom, which is not an indicator for the diagnosis of GPA; therefore, it was difficult to consider it in the differential diagnosis.

However, we affirmed that this case was atypical because the contrast-enhanced CT scan showed multifocal abscess formation in the nasal septum. Due to this abnormality, we performed a biopsy for diagnostic purposes.

Of the five cases of nasal septal abscess confirmed by contrast-enhanced CT in previous studies, three were bacterial [10–13], one was fungal [13], and all four were monocular type. Meanwhile, the other cases caused by GPA showed multifocal findings, as in our case [14].

In conclusion, when an investigation reveals a multifocal nasal septal abscess, the possibility of GPA should be considered as an atypical case of infectious abscess formation.

## Data Availability

The data are not publicly available due to privacy and ethical restrictions.

## Supplementary Materials

Supplementary Materials Video 1. Intraoperative endoscopic findings. The nasal septal mucosa is biopsied from different sites on both sides to prevent nasal septal perforation (biopsied from low horizontal on the left and high horizontal on the right septum membrane). A concha bullosa is also biopsied as a relatively normal area.
